# Understanding the health risks in basic science medical students: a cross sectional survey

**DOI:** 10.15694/mep.2020.000067.1

**Published:** 2020-04-10

**Authors:** Yogesh Acharya, Darien Merchant, Asia Manzoor, Jyothi Sathuluri, Anastasia Anoshina, Sateesh Babu Arja

**Affiliations:** 1 University Hospital; 2 Avalon University School of Medicine

**Keywords:** Medical Students, Medical Education, Health, Risk

## Abstract

This article was migrated. The article was marked as recommended.

Background

Medical students are usually subjected to a high workload environment and stress is one of the most important health risks that medical students encounter. The negative impact of stress on the student’s mental and general health in basic science has often been under reported.

Methods and Materials

A cross-sectional questionnaire survey was performed in Avalon University School of Medicine (AUSOM), Curacao, amongst the first to fourth semester basic science students with an objective to explore and understand their perspectives on different health risks.

**Results**More than ⅔ of the students **(**79.61%, n=82) were feeling stressed out during their basic sciences. The mean stress level among the basic science students was 7.42 ± 2.13 (scale:1-10). Common health issues encountered by the students were: sleep problems, impaired concentration, low mood, mood swings, difficulty in making decisions, emotional distress, anxiety, substance abuse, and abnormal weight gain.

Conclusion

Although lack of sleep and behavioral problems are the most common health issues encountered by the pre-clinical medical students. There is an urgent need to implement health promotion strategies in medical curriculum for mental and physical well-being of the students.

## Introduction

Medical school is a stressful time for physicians’ training. Medical students’ workload is considerably higher than that of many other non-medical students. Medical training has been recognized to account for numerous stressors that can directly affect students’ well-being. Academic pressures include issues, such as overwhelming burden of knowledge, different learning styles and the impact of the learning environment ( Dunn, Iglewicz and Moutier, 2008; Firth-Cozens, 2001; Tyssen *et al*., 2000). Due to the associated stressors, medical students have higher risks of developing certain health conditions such as obesity, stress, anxiety, fatigue, and depression ( Voltmer, Kötter and Spahn, 2012).

## Methods

The aim of our study was to explore and understand the different health risks in medical students. A cross-sectional survey was conducted at Avalon University School of Medicine (AUSOM), Curacao among 103 students of basic science semester MD1 - MD4 during September - December, 2018 semester. Potential participants were informed about the purpose of the study. Only the full-time basic science students attending AUSOM with more than 90% mandatory attendance as per the university attendance regulation were included in the study. Students who declined to be part of the study and were below the required attendance were excluded.

A customary set of questionnaires were organized and distributed into quantitative and qualitative questions **( Appendix 1 Questionnaire)**. Self-reports collecting social demographic data, including gender and current semester were taken into consideration. Questionnaires were gathered, analyzed and recorded after the completion. Stata 15© was used for data analysis. Quantitative data was represented with the help of mean, median, range, standard deviations, as applicable. The standard student’s t-test was applied to test the level of significance for two different groups. P<0.05 as considered to be statistically significant. Faculty senate validated by questionnaires, while the reliability estimated with the Cronbach’s alpha (.87) after the data collection.

Ethical Consideration

Approval for the study was obtained from the research and ethics committee of Avalon University. All the participants involved in this cross-sectional survey were aware of the purpose of this study. Student anonymity was upheld with top priority to ensure confidentiality of the study participants. Data were coded and stored in the university computer and protected with password for the authorized personnel to retrieve and analyze.

## Results/Analysis

One-hundred three, out of 105 students, participated in the study from MD1-MD4. More than ⅔ of the students (79.61%, n=82) reported feeling stressed out during basic science studies. Similarly, 43.69% (n=45) of the female and 56.31% (n=58) of the male respondents thought that medical students have a higher risk of health problems than other graduate programs. The mean stress level during the basic science studies was 7.42 ± 2.13 (scale:1-10). While, the mean stress during the examination time was 8.06 ± 2.04 (scale:1-10). The mean stress during the examination time was significantly higher than the average stress during the basic science (mean difference: -0.640 ± 0.288SE; 95% CI -1.2074 to -0.0726; P = 0.0273).

The most common health issues faced by medical students were: sleep problems (16.50%, n=17), impaired concentration (15.53%, n=16), low mood (13.59%, n=14), mood swings (12.62%, n=13), difficulty in making decisions (10.68%, n=11), emotional distress (9.71%, n=10), anxiety (6.80%, n=7), substance abuse (4.85%, n=5), abnormal weight gain (3.88%, n=4), and others not specified (2.91%, n=3). When asked about the ways to endure up with the academic pressure, the majority of students favored watching movies/TV/videos and listening to music (60%), sleeping (30%), exercising (7%), and other various activities (3%).

Regarding the sleep-hour, 6% of students slept 2-4 hours, 26% slept 4-6 hours, 14.5% slept 6-10 hours, and 2% slept 8-10 hours. Number of sleep-hour was compared between male and female students. Results showed that 31% of males and 21% females were getting 4-6 hours of sleep (difference:10%; 95% CI: -1.8810% to 21.5293%; P = 0.0994), and 21% of males and 8% females students were getting 6-10 hours of sleep (difference: 13%; 95% CI: 3.46 to 22.54%; P = 0.0076) respectively.

## Discussion

Medical training requires processes and procedures that differ significantly from other courses. High expectations and extensive knowledge-based learning coupled with rigorous clinical training requirements exposes medical students to high levels of stress and other related health problems ( Dahlin, Joneborg and Runeson, 2005). A study performed amongst the US and Canadian medical students ( Dyrbye, Thomas and Shanafelt, 2006) found high prevalence of depression and anxiety among medical students with higher overall psychological distress than the general population and age-matched peers. Similarly, incidence of burnout among residents and staff physicians is significantly higher than other professions ( Glauser, 2017). In the baseline survey of our study on medical student’s health, we found that most medical students were feeling stressed out in their basic science studies. Sleep problems, impaired concentration, low mood, mood swings, difficulty in making decisions, emotional distress, anxiety, and substance abuse was one of the key findings of our survey, and most of it is likely to be as a result of the academic pressure that each medical student needs to face every day.

In our study, male students had significantly higher sleep-hour than their female counterparts, although it did not achieve statistical significance. Similar to our study, a systematic review showed no significant difference between the depression rate between male and female students ( Cuttilan, Sayampanathan and Ho, 2016). However, in our sample male students were at high risk of having health problems compared to female students. Similarly, another study ( Giri, Baviskar and Phalke, 2003) concluded that sleep quality in female students is better than the males. Students with daytime sleepiness were associated with higher prevalence of addictions in males. Similarly, obesity is a growing problem among medical students ( Rampal *et al*., 2007; Abbate *et al*., 2006; Kumar and Ramaiah, 2005). Many medical students suffer from obesity due to the sedentary lifestyle, which makes them at greater risk of developing cardiovascular diseases and blood pressure conditions ( Bertsias *et al*., 2003). A study showed that overall, around ⅔ of the students experienced weight gain in the first year of university ( De Vos *et al*., 2015). Only 4.85% of students reported abnormal weight gain in our study, possibly due to our sample representing the first two years of medical studies.

Stress has been found to impair both mental and physical health, greater the stress the greater the impairment ( Sadeghi *et al*., 2018; Toussaint *et al*., 2014). Stress free lifestyle is known to have a major impact on the general health and academic performance of medical students. Beyond doubt, stress in medical school is likely to predict mental health problems later in life. But medical students seldom seek medical attention for their problems ( Abdulghani *et al*., 2011). It is imperative to find out the health risks in medical students to address them effectively ( Glauser, 2017). Many medical schools have started initiatives to reduce health risks in medical students through personal stress management programs, provision of support groups, organization of workshops, and formation of well-being committees and other psychosocial support ( Yiu, 2005). We recommend that medical schools should acknowledge these substantial health risks in medical students and formulate plans and policies to integrate systematic programs, early in curriculum, to decrease their health burden.

At Avalon University, there is help for students who are going through stress and personal issues by psychology counselor. Students can approach the counselor if they have any issues. At orientation, students are introduced to the school’s professional psychological counsellor program for an individual or family counselling including, but not limited to, depression, anxiety, and substance abuse. To maintain confidentiality, the student can directly contact the counsellor or may ask the help of the secretary, if desired, without the involvement of the faculty. At orientation, the student affairs office introduces students to the importance of recognizing the physical and emotional demands of their education on themselves as well as family. Students are provided information about stress management, relaxation, and realistic expectations for meeting the demands of medical school. The student affairs office focuses on issues such as stress management, communication, handling conflict, and other issues identified by students as important to their adjustment and well-being in medical school during student meetings.

In addition to psychology counselor, students are supported by student support programs like faculty mentorship program and peer mentoring program. AUSOM will provide a student mentor program through the Student Government Association (SGA). The student mentor program provides students with a great opportunity and valuable experience to interact with their peers as well as receive help in their physical and emotional adjustment to the medical school. It also aids in developing leadership and social skills of students. All new basic science students are assigned a student mentor. The student-mentor serves as a big brother/big sister to the new student to help in their personal life. The student-mentor benefits from the opportunity to develop leadership and social skills.

This study was conducted at AUSOM to operate as a model for a large-scale study, so the results are limited due to small sample size for general extrapolation in medical students. Despite these limits, the use of a validated set of questionnaire permits us to associate our findings to further studies done under similar situations and using the same evaluating mode. We recommend further comprehensive studies to be carried out through health organizations all over the world to report this critical concern of medical education.

## Conclusion

Sleep problems, impaired concentration, low mood, mood swings, difficulty in making decisions, emotional distress, anxiety, and substance abuse were major health problems reported by pre-clinical medical students. Psychological and social support, and regular counseling can be crucial to uplift general health of medical students. Medical schools should acknowledge the presence of substantial health problems in medical students and develop strategies to effectively address them.

## Take Home Messages

• Stress and other health issues are prevalent among the basic sciences students than expected.
• Even though stress and other mental health issues are common among medical students they do no seek help at an appropriate time.
• Medical schools should implement appropriate and adequate programs for the well-being of medical students.

## Notes On Contributors

Yogesh Acharya, M.D., FAcadMEd, Western Vascular Institute, Department of Vascular and Endovascular Surgery, University Hospital, National University of Ireland, Galway, Ireland.

Darien Merchant, Student, Avalon University School of Medicine, Willemstad, Curacao, Netherland Antilles.

Asia Manzoor, Student, Avalon University School of Medicine, Willemstad, Curacao, Netherland Antilles.

Jyothi Sathuluri, Student, Avalon University School of Medicine, Willemstad, Curacao, Netherland Antilles.

Anastasia Anoshina, Student, Avalon University School of Medicine, Willemstad, Curacao, Netherland Antilles.

Sateesh Babu Arja, M.B.B.S., MHPE, MSPH, FAMEE, SFHEA, FIAMSE, FAcadMEd, Associate Professor of Clinical Skills, Avalon University School of Medicine, Willemstad, Curacao, Netherlands Antilles.

## Appendices

### Appendix 1 Questionnaire

Age: Sex: M/F Class: MD1/MD2/MD3/MD4

Topic: Improving the understanding of health risks faced by medical students

1. Do you feel stress out in your basic science studies? (Yes/No) _______, Scale (1-10) _______

2. Do you think medical students have higher risk of health problems? (Yes/No) _______, Scale (1-10) ______

If yes, what can be different health problems in medical students? ____________________________________

3. How do you cope with your academic pressure? ________________________________________________

4. Do you have any health problem, which you consider to have a negative impact on your learning?

If yes, please describe it: _____________________________________________________________________

5. Did you experience any of these health issues during your basic science studies? (Yes/No) ____

a. Emotional distress b. Mood swings

c. Anxiety issues d. Difficulty in making decision

e. Concentration and memory impairment f. Sleep problems

g. Abnormal weight changes h. Substance abuse

i. Others (not specified here) ____________

6. How many hours of sleep do you get every night?

a. 2-4hrs b. 4-6hrs c. 6-8hrs d. 8-10hrs e. other:__________

7. How would you rate your stress during examination on a scale of 1-10?)__________

8. What are the most useful measures to decrease your stress?

a. Eating/overeating b. Watching movies/entertainment programs

c. Sleep d. Meditation

e. Exercise f. Smoking/Alcohol

g. Other: _______

9. Are you taking any prescribed drugs to cope with the academic pressure? (Yes/No) ______

10. What are your suggestions in improving the health of students faced with constant academic pressure?
