# Supplementary material for: Understanding the health risks in basic science medical students: a cross sectional survey
Source: MedEdPublish (2016). 2020 Apr 10;9:67. [Version 1] doi: 10.15694/mep.2020.000067.1 (PMC10697508; doi:10.15694/mep.2020.000067.1)
Supplement: Peer review history

# Reviewer report: Reviewer response for version 1

**DOI:** 10.21956/mep.20015.r30524

**Published:** 2020-04-12

**Authors:** Paolo Colet

**Affiliations:** 1 Nazarbayev University

This review has been migrated. The reviewer awarded 4 stars out of 5 This is an interesting study to read about medical students’ welfare. It is evident to me that the authors, too, did lots of work for the paper to get done. Below are my comments, and I hope the authors and readers would find it helpful.The abstract consists of essential parts that the reader finds it informative before reading the whole paper.The authors presented an introduction to contextualize the issues on medical student’s health risk and with the literature. However, I had trouble understanding the variables involved, especially the “health risk,” wherein part, the introductory text is about stress, or was it implied that the health risk is stress as stated in the abstract. Perhaps a discussion of the variable “health risk” was included to aid the reader. While the introduction is short, a statement or information on how the curriculum is set up at the Avalon University School of Medicine would help understand the context where the study was conducted. On methods, considering the aim of the study, which is to “explore and understand…” I wish a qualitative design or a mixed-method would have been used to address the aim. A bit more discussion on how the questionnaire was developed and of the validation process, including the language used, would also be helpful here. Also, I’m not sure if the data collection was through paper and pencil or online. About the data analysis, I wish there was also a statement on how the data analysis for the open-ended questions was handled. The authors addressed the ethical consideration in the study; however, no information about the process of obtaining consent was provided or was it implied when the authors mentioned that students were aware of the study.On the results, the presentation started with the respondent’s characteristics and followed by the data obtained from the questionnaire; however, I cannot see where the results of the open-ended questions were. Also, I wish they included a table to display the respondents’ characteristics and of the descriptive statistics of the variable under study.In the discussion, the authors provided a detailed analysis of their findings consistent with previous research. The first paragraph contains a summary of the findings, which gives the reader a general view. Also, the authors interpreted the result where there was no statistical significance was achieved between males and females on “sleep-hour,” and supported by the literature. The discussion about psychological counseling at Avalon University would have been presented earlier in the introduction of the study. The recommendation, conclusion, and take-home messages are rightfully in place.Overall this paper is valuable to stakeholders, educators, and students.

Reviewer Expertise:

NA

No decision status is available

# Reviewer report: Reviewer response for version 1

**DOI:** 10.21956/mep.20015.r30526

**Published:** 2020-04-12

**Authors:** P Ravi Shankar

**Affiliations:** 1 American International Medical University

This review has been migrated. The reviewer awarded 4 stars out of 5 Thank you for the invitation to review this interesting paper. I am happy to note that many of the co-authors are medical students. The article is generally well-written though language and grammar corrections may be required in a few places. I am not sure about the title of the manuscript. I think health risks may be OK but the first thing which comes to my mind on hearing the phrase is infections like hepatitis B and HIV/AIDS. How many students could not participate in the study because their attendance was below the 90% required for participation? Why did the authors choose this cut off for participation? I think the participation rate is very good. Where was the questionnaire administered? The authors can explain in greater detail which parameters were compared among different subgroups of respondents? Did these variables follow a normal distribution? The Introduction is very brief and the authors can consider expanding the same. Toward the end of the Introduction they can provide a brief explanation of the study and highlight the study objectives. The Discussion is well written and the authors review the student support systems present in their institution. I did not understand the Acknowledgements section. The Appendix shows the questionnaire used in the study. How was the questionnaire developed? How was it validated? It will be interesting if the study can also be conducted among clinical students. This is an important study and will be of interest to a wide group of medical educators.

Reviewer Expertise:

NA

No decision status is available

# Reviewer report: Reviewer response for version 1

**DOI:** 10.21956/mep.20015.r30525

**Published:** 2020-04-11

**Authors:** Deb Halder

This review has been migrated. The reviewer awarded 4 stars out of 5 Notes on the Abstract, Reference and AppendicesThe abstract has categorically dealt with the tools and techniques which are used in this study in brief. The references are highly relevant to the scope of the paper and recent studies have been taken for evaluating the study. The questionnaire reveals mixed in nature, combination of closed ended and open ended questions. Kim (1970) established a stress scale to measure the academic stress of the tertiary level of learners. This questionnaire could have been highly significant if it could be more structured and organic.IntroductionIntroduction cannot highlight what the paper wants to study; rather it exposed some academic difficulties a student experiences. MethodologyThis part could have been more justified why these methods were applied, why triangulation of both quantitative and qualitative has been necessitated.Discussion The best part of this paper is its discussion which has been comparatively analyzed with the references of reasonable studies. But the portion beginning with “We recommend that medical schools should acknowledge these substantial health risks in medical students and formulate plans and policies to integrate systematic programs, early in curriculum, to decrease their health burden” cannot fit with the discussion; rather, a different part of recommendation suits this validated assumption.Overall, the paper is readable and reliable with its findings.

Reviewer Expertise:

NA

No decision status is available

# Reviewer report: Reviewer response for version 1

**DOI:** 10.21956/mep.20015.r30523

**Published:** 2020-04-10

**Authors:** Felix Silwimba

**Affiliations:** 1 University of Lusaka

This review has been migrated. The reviewer awarded 5 stars out of 5 this is a very good study in medical education. it partly explains some experiences observed in qualified doctors in low income countries. I recommend this study.

Reviewer Expertise:

NA

No decision status is available
